# Supplementary material for: Dental age prediction from panoramic radiographs using machine learning techniques
Source: PLOS Digit Health. 2025 Oct 30;4(10):e0001077. doi: 10.1371/journal.pdig.0001077 (PMC12574863; doi:10.1371/journal.pdig.0001077)
Supplement: S1 File — (DOCX) [file pdig.0001077.s001.docx]

# TRIPOD-AI Checklist

# Dental age prediction from panoramic radiographs using machine learning techniques

| TRIPOD-AI Item | Covered in Manuscript (Yes/No) | Location / Notes |
| --- | --- | --- |
| Title | Yes | Title page: 'Dental age prediction from panoramic radiographs using machine learning techniques' |
| Abstract | Yes | Abstract includes objectives, methods, dataset (550 images), model (YOLOv11), results (accuracy), and conclusion |
| Background and Objectives | Yes | Introduction section, background on DA estimation, rationale for AI use, study objectives outlined |
| Source of Data | Yes | Materials and Methods: dataset of 550 panoramic radiographs, ages 3–14 |
| Participants | Yes | Materials and Methods: children 3–14, dataset description, inclusion/exclusion criteria (poor quality excluded) |
| Outcome to be Predicted | Yes | Defined as dental age groups based on AAPD reference chart |
| Predictors | Yes | Radiographic features (panoramic images), extracted automatically by YOLOv11 model |
| Sample Size | Yes | Dataset: 550 images (train/val) and 203 test images; limitations discussed in Discussion |
| Missing Data | No | Not specifically described how missing data was handled beyond exclusion of poor-quality radiographs |
| Statistical Analysis Methods | Yes | Training procedure, augmentation, optimizer, performance metrics (Top-1, Top-5), Cohen’s kappa |
| Model Development | Yes | YOLOv11 architecture and training details in Methods |
| Model Specification | Yes | Detailed description of model structure, hyperparameters, architecture |
| Model Performance | Yes | Results: validation/test accuracy, confusion matrix, Grad-CAM visualizations |
| Model Explainability | Yes | Grad-CAM used for model interpretability |
| Results: Participants | Yes | Table 1 distribution, total number of images included |
| Results: Model Performance | Yes | Validation and test performance reported with metrics |
| Limitations | Yes | Discussion: dataset size, imbalance, single center, need for multicenter validation |
| Interpretation | Yes | Discussion and Conclusion: clinical implications, potential translation into practice |
| Implications for Practice | Yes | Discussion: usefulness in pediatric dental practice, cost–benefit |
| Supplementary Information | Partially | Figures provided (confusion matrix, Grad-CAM). Checklist itself now added as supplementary material. |
| Funding | No | Funding statement not explicitly mentioned in current text |
| Conflict of Interest | Yes | Declared at the end: None declared |
| Acknowledgements | Yes | Acknowledgement section present |
