# Supplementary material for: Dental age prediction from panoramic radiographs using machine learning techniques
Source: PLOS Digit Health. 2025 Oct 30;4(10):e0001077. doi: 10.1371/journal.pdig.0001077 (PMC12574863; doi:10.1371/journal.pdig.0001077)
Supplement: Attachment — Submitted filename: Dental Age (Response to Reviewers).docx [file pdig.0001077.s003.docx]

PLOS Digital Health

Manuscript ID: PDIG-D-25-00375

Title: Dental age prediction from panoramic radiographs using machine learning techniques

Dear Dr. Leo Anthony Celi,

We sincerely thank you for the opportunity to revise our manuscript and for the valuable comments provided. We have carefully addressed all the points raised, and the revisions have improved the overall quality of the paper. As requested, we are submitting the revised files in the required formats: one version with track changes and one clean version. These files are being submitted together with our detailed response to the reviewers. We hope that the revised manuscript will now be suitable for acceptance. Please do not hesitate to contact us should any further clarifications be needed.

Dr. Mehdi Salehizeinabadi, on behalf of all authors

**Journal Requirements:**

1. Please ensure that your Ethics Statement is available in its entirety at the beginning of your Methods section, under a subheading 'Ethics Statement'.

Thank you for your comment. The Ethics Statement has been reviewed and updated as required. It is now placed at the beginning of the Methods section, under the subheading “Ethics Statement”, and reads as follows:

***“Ethics Statement***
*This study was reviewed and approved by the University of Alberta Research Ethics Board (REB) under the ethics ID Pro00143202. The panoramic radiographs were obtained from the Oral and Maxillofacial Radiology service and were de-identified prior to analysis. Because the dataset consisted of historical clinical radiographs without any personal health identifiers, and no contact with patients was required, the University of Alberta REB waived the requirement for informed consent.”*

2. Please upload separate figure files in .tif or .eps format. Also, remove the figures from your manuscript file but keep the legends.
For more information about figure files please see our guidelines: <https://journals.plos.org/digitalhealth/s/figures>
https://journals.plos.org/digitalhealth/s/figures#loc-file-requirements

Thank you for your comment. As requested, I have removed the figures from the main manuscript file while keeping the legends. The figures have been uploaded separately in the required .tif/.eps formats according to the journal’s guidelines.

3. In the online submission form, you indicated that “ The data that support the findings of this study are available from the University of Alberta but restrictions apply to the availability of these data, which were used under license for the current study, and so are not publicly available. Data are however available at dentrsch@ualberta.ca upon reasonable request and with permission of University of Alberta.”.

All PLOS journals now require all data underlying the findings described in their manuscript to be freely available to other researchers, either
1. In a public repository,
2. Within the manuscript itself, or
3. Uploaded as supplementary information.

This policy applies to all data except where public deposition would breach compliance with the protocol approved by your research ethics board. If your data cannot be made publicly available for ethical or legal reasons (e.g., public availability would compromise patient privacy), please explain your reasons by return email and your exemption request will be escalated to the editor for approval. Your exemption request will be handled independently and will not hold up the peer review process but will need to be resolved should your manuscript be accepted for publication. One of the Editorial team will then be in touch if there are any issues.

If the reviewer comments include a recommendation to cite specific previously published works, please review and evaluate these publications to determine whether they are relevant and should be cited. There is no requirement to cite these works unless the editor has indicated otherwise.

Dear Editorial Team,

Thank you for your message regarding the PLOS data sharing policy. I would like to formally request an exemption from making the dataset publicly available for our manuscript titled "Dental age prediction from panoramic radiographs using machine learning techniques" (Manuscript ID: PDIG-D-25-00375).

The data used in this study consist of panoramic radiographs and associated patient information obtained under strict privacy and confidentiality agreements with the University of Alberta. Access to these data is governed by the university’s Health Information privacy policies and is only granted to researchers who have completed mandatory privacy and security training and obtained the necessary institutional certifications.

As per the terms of our data access agreement, we are permitted to use these data solely for the approved study and cannot publicly share them in any form that could compromise patient confidentiality. Any data access requests must be reviewed and authorized directly by the University of Alberta. Interested researchers may contact the School of Dentistry Research Office at dentrsch@ualberta.ca to inquire about potential access, subject to institutional approval and compliance with privacy regulations.

All data handling procedures in our study complied with ethical approval and university policies, and the dataset remains securely stored within the institution’s protected servers.

**Additional Editor Comments (if provided):**

This manuscript has been evaluated based on one external peer review and my own assessment as Academic Editor. Below are the areas which I think should be prioritized in the revision:
1. Adding comparative analysis of different deep learning architectures as mentioned by the reviewer.
2. Adding the two key points regarding the Discussion and Study Limitations section mentioned by the reviewer.
3. Addressing the points mentioned for Results, Discussion and Recommendation section by the reviewer.
4. Addressing the points mentioned by myself as the reviewer.

We thank you for the careful assessment of our manuscript. We are pleased to confirm that all requested revisions have been addressed:

1) Comparative analysis of DL architectures: Added discussion on alternative models (ResNet, EfficientNet, DenseNet), rationale for choosing YOLOv11, and future benchmarking plans.

2) Discussion and Limitations: Expanded to cover dataset imbalance (reflecting real-world OPG patterns) and mitigation strategies (augmentation + class weights), with recommendations for future approaches.

3) Results/Discussion/Recommendations: Revised to detail misclassification patterns and clinical implications, corrected the regression wording, added a cost–benefit perspective, specified augmentation parameters, discussed outlier handling, clarified data/code availability, and noted calibration assessment as a limitation/future work.

4) Editorial points:All additional comments have been integrated for consistency across Methods, Results, Discussion, and Limitations.

**Reviewer #1:**

Thank you for sharing this article with me. I found it genuinely engaging. The study offers a thoughtful and well-organized approach to dental age estimation using deep learning, and the topic itself is clearly important for clinical practice in dentistry. Still, there are a few issues that the authors should address, along with some sections that would benefit from further clarification.

**Introduction**
1-It is recommended to address the challenges of dental age estimation based on current conventional methods in pediatric dentistry, along with their associated limitations and clinical sensitivities.
Thank you for this valuable feedback. In response, we have revised the Abstract and Introduction to address the challenges of conventional dental age estimation methods in pediatric dentistry. Specifically, we have added the following sentence to highlight their limitations:

*“Conventional methods for dental age estimation are limited by population dependency, inter-observer variability, and reduced reliability during mixed dentition phases, which can compromise accuracy and clinical sensitivity across diverse pediatric populations.”*

**Methods section**
2-Given that conventional machine learning methods are generally unsuitable for medical image processing, it is essential to clarify why simpler and more traditional machine learning algorithms were not employed despite the limited sample size, and why the authors opted for deep learning techniques after expanding the dataset, all while strictly adhering to scientific principles. Furthermore, the rationale for not comparing traditional machine learning methods with the implemented deep learning approach in this study must be explicitly explained. It is plausible that conventional machine learning models could achieve accuracy levels comparable to the proposed model, which should then be thoroughly analyzed and discussed in the Discussion section.

Thank you for this thoughtful and important comment. We agree that clarifying our rationale for model selection is essential, particularly given the modest sample size.

We chose not to implement traditional machine learning (ML) methods such as support vector machines (SVMs), k-nearest neighbors (k-NN), or random forests for several technical and scientific reasons. Panoramic radiographs contain high-dimensional and spatially complex information. Traditional ML requires handcrafted feature extraction prior to classification, which introduces observer bias and often limits performance when subtle anatomical variations are critical for prediction. By contrast, deep learning (DL) models—particularly convolutional architectures such as YOLOv11—can automatically learn hierarchical features directly from raw images, capturing intricate developmental patterns that are difficult to engineer manually.

Recent studies reinforce this distinction. For example, Vila-Blanco et al. demonstrated that CNN-based approaches achieved higher accuracy and generalizability than ML algorithms in dental age estimation. Similarly, Kim et al. and Roh et al. reported that traditional ML models plateaued in performance despite extensive preprocessing, whereas DL models-maintained accuracy when trained on raw radiographs.

Furthermore, our decision to use YOLOv11 was supported by augmentation and generalization strategies that helped address the limited dataset. While traditional ML may perform adequately on structured/tabular data, it is less robust when applied to raw image data without dimensionality reduction or engineered features, steps that could reduce clinical applicability. YOLOv11 was also chosen for its demonstrated success in related dental applications, including caries detection and surgical risk assessment, showing strong precision and efficiency even in moderate datasets.

We acknowledge that a direct comparison between DL and traditional ML could provide additional insights. However, such an analysis was beyond the scope of this study, which aimed to evaluate the feasibility of a fully automated DL approach for clinical integration. We have now added a clarification in the Discussion section to explain this rationale and highlight comparative benchmarking with traditional ML as an important avenue for future research:

*“We did not include comparisons with conventional machine learning methods (e.g., SVM, k-NN, random forests) because these approaches typically require handcrafted feature extraction, which introduces observer bias and limits performance in high-dimensional image data. In contrast, deep learning models such as YOLOv11 can automatically learn clinically relevant features directly from raw panoramic radiographs, an advantage highlighted in recent studies. Moreover, traditional ML approaches have shown limited scalability in dental imaging tasks despite feature engineering, whereas DL models maintain accuracy with raw input. While a direct comparison would be scientifically valuable, it was beyond the scope of the present study, which focused on evaluating the feasibility of a fully automated DL pipeline. We propose this as a direction for future research.”*

3-Including a comparative analysis of different deep learning architectures—like CNN, ResNet, and EfficientNet—would significantly strengthen the paper and clarify the findings for readers. Without such a comparison between various DL-based approaches, the current study leaves room for uncertainty about whether alternative methods might yield different results. Addressing this gap would enhance the paper’s overall value and credibility.

Thank you for this constructive suggestion. We fully acknowledge that including a comparative analysis of different deep learning (DL) architectures—such as ResNet, EfficientNet, and other CNN-based models—would provide valuable context for evaluating the performance of our YOLOv11-based approach.

In this study, we deliberately focused on YOLOv11 because of its recent architectural enhancements that make it highly suitable for medical imaging tasks requiring both classification and localization, while maintaining a balance between predictive accuracy and computational efficiency. YOLOv11 incorporates attention modules (C2PSA) and compact convolutional blocks (C3k2), which enable effective learning from relatively modest datasets by enhancing feature extraction and focusing on salient image regions. Recent studies have demonstrated its superior performance in dental imaging applications, such as automated detection of caries and assessment of third molar extraction difficulty, further supporting its selection in our work.

While a head-to-head comparison with other DL architectures would certainly strengthen the study, such an analysis was beyond the scope of the current project. We have now added a statement in the Discussion section to acknowledge this limitation and highlight the importance of future studies that benchmark YOLOv11 against other state-of-the-art DL models (e.g., ResNet, EfficientNet, DenseNet) under standardized conditions to validate and extend our findings:

*“Although this study focused exclusively on YOLOv11, we acknowledge that comparative evaluation with other deep learning architectures, such as ResNet, EfficientNet, or DenseNet, would provide additional insights into relative performance. Our choice of YOLOv11 was based on its recent architectural improvements, including C2PSA and C3k2 modules, which enhance feature learning and efficiency in medical imaging tasks. Furthermore, YOLOv11 has demonstrated strong results in dental applications, including caries detection and surgical risk assessment. Future work should directly benchmark YOLOv11 against other CNN-based models under standardized preprocessing and evaluation pipelines to validate its comparative advantages.”*

4-Dataset Augmentation and Preprocessing section shows:
The paper mentions how the images are distributed across different age groups. A table or a chart displaying the class distribution would be better than text to show the existing imbalance because it would visually demonstrate the problem. Visualizing the data allows readers to better grasp the problem of unbalanced data distribution.

Thank you for this helpful suggestion. In response, we have added a bar chart to visually represent the distribution of images across the 11 dental age groups. This visualization clearly highlights the class imbalance in our dataset, particularly in the younger and older age groups, which we have further discussed in the revised manuscript. We agree that visualizing this distribution allows readers to better understand the potential challenges for model training and classification performance.

| Age Group (Years) | Number of Images (%) |
| --- | --- |
| 3–4 | 17 (3.1) |
| 4–5 | 15 (2.7) |
| 5–6 | 19 (3.5) |
| 6–7 | 28 (5.1) |
| 7–8 | 41 (7.5) |
| 8–9 | 66 (12.1) |
| 9–10 | 83 (15.2) |
| 10–11 | 75 (13.7) |
| 11–12 | 71 (13.0) |
| 12–13 | 66 (12.1) |
| 13–14 | 69 (12.6) |
| Total | 570 (100) |

Table 1. Distribution of panoramic radiographs across the 11 age groups.

The paper should address two key points regarding the Discussion and Study Limitations section in the following manner:
1. The negative effect of sample imbalance on model performance for age groups with limited data should be discussed.

Thank you for highlighting the importance of discussing the impact of sample imbalance on model performance. In our dataset, the imbalance across age groups reflects the real-world distribution of orthopantomogram (OPG) requests in clinical practice. For example, younger age groups (particularly those under 8 years) are less frequently referred for OPGs due to both clinical guidelines and practical considerations, resulting in naturally fewer available cases for these categories. Thus, while imbalance is present, it represents authentic clinical demand rather than artificial sampling bias.

We also acknowledge that the overall dataset size was relatively modest. Despite these constraints, our YOLOv11-based model achieved a high classification accuracy of 92.6%, suggesting that it was able to generalize effectively and maintain robust performance even in underrepresented age groups. This outcome highlights the model’s resilience but also underscores the importance of future work using larger, more balanced, multi-institutional datasets to validate and extend these findings. We have now incorporated this point into the Discussion/Study Limitations section of the revised manuscript:

*“In addition, the dataset exhibited imbalance across age groups, which may have influenced predictive performance for categories with fewer samples. This imbalance reflects real-world clinical patterns, as OPGs are less frequently indicated in younger children due to clinical guidelines and practical considerations. Although our model maintained high overall accuracy despite these disparities, future studies should validate performance on larger, more balanced, multi-center datasets to confirm generalizability across all age groups.”*

2. The paper should explain whether it considered any specific strategies such as weighted loss functions or targeted data augmentation for minority classes to mitigate this issue.

We appreciate this valuable suggestion regarding strategies to address class imbalance. In the present study, we employed both targeted data augmentation and class weighting to mitigate the effects of underrepresented age groups. For minority classes, we applied augmentation techniques such as random rotations (±15°), translations (up to 10%), and brightness/contrast variations (±20%) to increase sample diversity and improve model generalization. Additionally, class weights were incorporated into the loss function, ensuring that underrepresented categories contributed proportionally more during training and reducing bias toward majority age groups.

These combined strategies helped the model maintain high overall accuracy despite the inherent imbalance in the dataset. We acknowledge, however, that further exploration of advanced rebalancing methods (e.g., focal loss, synthetic oversampling, or ensemble approaches) could provide additional benefits. This point has now been added to the Methods and Study Limitations sections as a direction for future research:

*“To address class imbalance across age groups, class weights were applied within the loss function, ensuring that underrepresented categories contributed proportionally more to the optimization process. This adjustment reduced the bias toward majority classes and complemented our targeted augmentation strategies.” And:*

*“To mitigate the impact of class imbalance, we applied targeted augmentation to minority age groups (e.g., rotations, translations, brightness/contrast adjustments) and incorporated class weights into the loss function, allowing underrepresented categories to contribute more strongly to the optimization process. These strategies improved generalization across unevenly distributed classes. Nevertheless, future work should explore additional approaches, such as focal loss, oversampling, or ensemble methods, to further enhance performance in highly imbalanced datasets.”*

In the second paragraph on page 6, data augmentation techniques are mentioned. It is recommended to provide more specific parameters for these techniques (e.g., the exact ranges for brightness/contrast variation) to:
1. Enhance readers' understanding of the preprocessing pipeline, and
2. Improve the reproducibility of the study's results.
Thank you for this helpful recommendation. In the revised manuscript, we have now specified the exact ranges applied for brightness, contrast, rotation, translation, and random erasing:

*“To enhance generalizability and reduce the risk of overfitting, the training set subsequently underwent two rounds of data augmentation. Techniques included horizontal flipping (probability = 0.5), random rotations (±15°), translations (up to 10% of image dimensions), brightness variation (±20%), contrast variation (±20%), and random erasing with a probability of 0.4 (erasure size up to 20% of image area).”*

Results
1. Line 174:
The statement "A total of X panoramic radiographs was explored..." should either:
a) Replace X with the exact number of samples, or
b) Justify if the precise number cannot be disclosed.

Thank you for noting this issue. We have corrected the placeholder and clarified the dataset composition. The study included 753 unique panoramic radiographs (550 for training/validation and 203 for testing). The training set of 440 images was expanded to 1,320 images through augmentation, while the validation set (110 images) and test set (203 images) remained unaltered.

2. Lines 188-189:
The observation that "a few misclassifications were observed between adjacent age groups…" is worth more consideration. A few ideas to include more discussion might be:
• Patterns: Are misclassifications concentrated in specific age groups?
• Anatomical Correlates: Were misclassifications typical linked to any specific anatomical features?
• Mitigation Strategies: What can be done to reduce these errors in future research?
• Clinical implications:
o If clinically insignificant, explain how this supports the methodological approach.
o If clinically significant, provide a fuller discussion of implications.
Thank you for this thoughtful comment. We agree that the observed misclassifications between adjacent age groups merit further discussion. We have now expanded the Discussion to include the following considerations:

1) Patterns: Most misclassifications occurred between neighboring age groups (e.g., 7-8 vs. 8-9 years, or 11-12 vs. 12-13 years), which reflects the subtle developmental differences in dentition within short chronological intervals.

2) Anatomical Correlates: These errors were often linked to borderline cases in which molar eruption or root development overlapped across two consecutive categories, making classification inherently challenging even for human experts.

3) Mitigation Strategies: Future work could reduce these errors by using larger datasets to improve representation of transitional stages, exploring ordinal classification or regression-based approaches to account for the continuous nature of dental development, and integrating multi-modal data (e.g., combining panoramic radiographs with clinical records).

4) Clinical Implications: Importantly, these misclassifications are clinically insignificant, as they occurred only between adjacent groups that differ by less than one year. In practice, treatment planning decisions (e.g., space maintenance, interceptive orthodontics) are not affected by such small variations in estimated dental age. This supports the robustness and practical utility of our methodological approach.

We have clarified this concern in the Results and Discussion sections as below:

*“The confusion matrix in Figure 2 provides a detailed breakdown of the model’s predictions across all dental age groups. While overall accuracy was high, most misclassifications were concentrated between adjacent age categories (e.g., 7-8 vs. 8-9 years, and 11-12 vs. 12-13 years), reflecting the subtle developmental changes that occur in close chronological intervals.” And,*

*“Closer examination showed that these errors were typically associated with borderline anatomical features such as overlapping stages of molar eruption and root development, which are difficult to distinguish even for expert clinicians. This suggests that the misclassifications reflect biological variability more than algorithmic shortcomings. To mitigate such errors, future work could benefit from larger datasets with greater representation of transitional stages, adoption of ordinal classification/regression frameworks, or incorporation of multimodal data (e.g., combining radiographs with clinical records). Clinically, these misclassifications are not significant because treatment planning decisions rarely depend on differences of less than one year in dental age. This supports the robustness and practical utility of our proposed approach despite minor variability at group boundaries.”*

Discussion:
(Lines 246-247): The statement "our model achieved higher precision via regression analysis and automated full-image processing" seems to contradict:
1. The paper's claim of "direct classification" (Line 221), and
2. The actual output format of the model, i.e., 11 discrete age categories.
We recommend the authors to:
• Clarify this methodological discrepancy or explain if regression analysis was ever included in the pipeline
Thank you for pointing out this inconsistency. You are correct that our study employed a direct classification approach into 11 discrete age categories, not regression. The earlier statement suggesting “higher precision via regression analysis” was a miswording and has now been corrected to accurately reflect our methodology. Our model output was categorical classification, consistent with the description in the Methods and Results sections. We have clarified this in Discussion:

*“Unlike the quadrant-based approach of Kurniawan et al. [46], which required manual cropping and reported 74% accuracy through classification, our model achieved higher precision through automated full-image classification without the need for manual cropping or regression-based pipelines.”*

Lines 289-290: The phrase "restricted to the pediatric population aged from X to Y" calls for clarification.

Thank you for the comment. We have clarified the age range.

Recommendation:
As a recommendation, one should consider carrying out cost-benefit analyses to create further clinical relevance for this study. In particular, the discussion should be framed in terms of the following points:
1. Operational Efficiency:
How the proposed model reduces time and resource expenditures for dental age estimation when compared to conventional methods
2. Clinical Impact:
Possible reduction of diagnostic errors
Increase in the quality of patient care
3. Implementation Value:
Cost-effectiveness of implementation of this AI in the clinical setting
Compare with manual assessment workstreams

Thank you for this valuable recommendation. We agree that a cost–benefit perspective enhances the clinical relevance of our findings. In the revised manuscript, we have expanded the Discussion to highlight operational efficiency, clinical impact, and implementation value of the proposed approach, particularly in comparison with conventional manual methods of dental age estimation:

*“From a cost–benefit perspective, the proposed YOLOv11-based model offers several advantages over conventional manual methods of dental age estimation. First, in terms of operational efficiency, the model delivers automated predictions within seconds, reducing the need for time-intensive expert staging and interpretation of panoramic radiographs. This efficiency may lower resource expenditures and increase throughput in busy clinical or forensic settings. Second, the clinical impact is notable: automation has the potential to minimize diagnostic variability and reduce human error, thereby improving the consistency and quality of patient care. Third, from an implementation standpoint, the integration of an AI-based system into existing digital radiography workflows is likely to be cost-effective compared with repeated manual assessments by specialists, especially in resource-limited environments. While a full economic evaluation was beyond the scope of this study, these benefits suggest that the model could enhance accessibility and scalability of dental age estimation in clinical practice. Future work should incorporate formal cost–benefit analyses to quantify these advantages and provide additional evidence to guide adoption.”*

**Reviewer #2:**

The paper addresses an important topic with a novel application for pediatric dental age classification. However, there are certain areas where the manuscript can be improved utilizing the TRIPOD +AI checklist:
1. Dataset and Code availability: Both are not provided, which diminishes reproducibility of the research.

We appreciate the reviewer’s comment on reproducibility. Due to privacy and confidentiality restrictions imposed by the University of Alberta, the dataset of panoramic radiographs cannot be publicly shared. All data handling was conducted under institutional ethics approval, and the dataset remains securely stored on protected university servers. We have clarified this in the revised manuscript and included a Data Availability statement, providing a pathway for interested researchers to request access subject to institutional approval. The code used for model development will be made available upon reasonable request.

**Data Availability**
The data used in this study consist of panoramic radiographs and associated patient information obtained under strict privacy and confidentiality agreements with the University of Alberta. Access to these data is governed by the University’s Health Information privacy policies and is only granted to researchers who have completed mandatory privacy and security training and obtained the necessary institutional certifications.

As per the terms of our data access agreement, the dataset cannot be publicly shared. Interested researchers may contact the School of Dentistry Research Office at dentrsch@ualberta.ca to inquire about potential access, subject to institutional approval and compliance with privacy regulations. All data handling procedures in this study complied with ethical approval (Pro00143202) and university policies, and the dataset remains securely stored within the institution’s protected servers.

**Code Availability**
The code used to preprocess the data, train the YOLOv11 model, and perform analyses is available from the corresponding author upon reasonable request.

2. Outliers: There is no discussion about outliers. Although outliers are typically for numerical data, mentioning it would be helpful.

Thank you for raising this point. While outliers in the traditional statistical sense are less common in image-based datasets, we recognize the importance of clarifying how potential outlier cases were handled. In this study, outliers primarily took the form of low-quality radiographs (e.g., images with artifacts, distortions, or overlapping anatomical structures). These cases were systematically excluded during the preprocessing stage to ensure dataset consistency and to minimize the risk of biasing model performance. We have now added a brief discussion of this point to the revised manuscript IN Methods and Discussion sections:

*“To ensure consistency and quality, radiographs with significant artifacts, low resolution, or overlapping anatomical structures were excluded. These cases were considered potential outliers, as their inclusion could disproportionately influence model learning, and were removed during preprocessing.” And,*

*“Another point to note is the potential influence of outlier cases. In this study, outliers were primarily radiographs of insufficient quality or with significant artifacts, which were excluded during preprocessing. While this ensured a consistent dataset, future work could explore automated quality-control pipelines to systematically identify and manage such outliers in larger multi-institutional datasets.”*

3. Calibration performance: The model’s performance should also be reported for calibration.

Thank you for this important comment. We agree that calibration is a critical aspect of model evaluation, particularly for clinical translation. In the current study, we focused on classification accuracy (Top-1 and Top-5) and generalizability across an independent test set. We did not perform a formal calibration analysis (e.g., reliability diagrams, Brier score, or expected calibration error). We acknowledge this as a limitation and will consider incorporating calibration metrics in future work to better assess the agreement between predicted probabilities and true outcomes, which would further support the model’s reliability for clinical use. This has now been noted in the revised manuscript in Discussion:

*“Another limitation of this study is the absence of a formal calibration analysis. While the model demonstrated high classification accuracy, calibration metrics such as reliability diagrams, Brier scores, or expected calibration error were not evaluated. Future studies should incorporate these measures to assess how well the predicted probabilities align with true outcomes, thereby strengthening confidence in the model’s clinical applicability.”*
