# Supplementary material for: Dental age prediction from panoramic radiographs using machine learning techniques
Source: PLOS Digit Health. 2025 Oct 30;4(10):e0001077. doi: 10.1371/journal.pdig.0001077 (PMC12574863; doi:10.1371/journal.pdig.0001077)
Supplement: Attachment — Submitted filename: Response to reviewer.docx [file pdig.0001077.s004.docx]

PLOS Digital Health

Manuscript ID: PDIG-D-25-00375

Title: Dental age prediction from panoramic radiographs using machine learning techniques

Dear Dr. Leo Anthony Celi

We are grateful for the opportunity to revise our manuscript and for the reviewers’ insightful feedback. We have carefully addressed all comments, and the revisions have strengthened the overall quality of the paper. As requested, we are submitting two versions of the manuscript—one with tracked changes and one clean copy—along with our detailed response to the reviewers. We hope that the revised submission will now meet the requirements for acceptance, and we would be happy to provide any further clarification if needed.

Dr. Mehdi Salehizeinabadi, on behalf of all authors

**Comments:**

1-Table 1 totals 570 panoramic radiographs, but Abstract and other sections mention 550 radiographs. If 20 were excluded, then it should be mentioned clearly.

We appreciate the reviewer’s careful observation regarding the inconsistency in the reported number of radiographs. The correct number of radiographs is **550**, as consistently stated in the Abstract and throughout the manuscript. We have corrected this typographical error in the final version of the manuscript, and the total is now correctly reported as 550.

2-Consider uploading a completed TRIPOD-AI checklist as Supplementary material and cross-referencing it in the Material and Methods section.

We thank the reviewer for this valuable suggestion. As recommended, we have completed the TRIPOD-AI checklist and provided it as Supplementary Material (Appendix 1). In addition, we have revised the Materials and Methods section with the following statement:

*In accordance with reporting standards for AI-based prediction models, the completed TRIPOD-AI checklist has been provided as Supplementary Material.*

3-**Figure resubmission:**

While revising your submission, we strongly recommend that you use PLOS’s NAAS tool (<https://ngplosjournals.pagemajik.ai/artanalysis>) to test your figure files. NAAS can convert your figure files to the TIFF file type and meet basic requirements (such as print size, resolution), or provide you with a report on issues that do not meet our requirements and that NAAS cannot fix.

We appreciate the recommendation. All figure files have been revised and resubmitted in accordance with the requirements announced by the journal, and we have ensured that they meet the specified standards.
